# Prescribing Patterns and Medication Appropriateness in General Medicine: Evaluation of Adherence to WHO Guidelines at a Tertiary Care Teaching Hospital in South Delhi, India

**DOI:** 10.7759/cureus.99580

**Published:** 2025-12-18

**Authors:** Shreshth Khanna, Ahmad Zee Fahem, Bhaskar Malik, Mayank Malik

**Affiliations:** 1 Pharmacology and Therapeutics, Hamdard Institute of Medical Sciences and Research, New Delhi, IND; 2 Pharmacology, Autonomous State Medical College, Gonda, IND; 3 Anesthesiology, Employees State Insurance Corporation (ESIC), Alwar, IND; 4 Pharmacology, Sanskaram Medical College, Jhajjar, IND

**Keywords:** antibiotic stewardship, national list of essential medicines, polypharmacy, prescription audit, rational prescribing, who prescribing indicators

## Abstract

Background: Rational prescribing is vital to ensure patient safety, reduce medication errors, and optimize healthcare resource utilization. Prescription audits serve as essential tools for evaluating and improving prescription practices in clinical settings.

Objectives: The objectives of the study are to assess the prescription patterns and the completeness of prescriptions in the outpatient department of a tertiary care teaching hospital, using the World Health Organization (WHO) core prescribing indicators, and to identify areas for improvement.

Methods: A retrospective observational study was conducted by analyzing 300 randomly selected printed prescriptions from the Department of Medicine over six months. Data were extracted on patient demographics, prescription completeness, drug categories, and adherence to WHO prescribing indicators. Descriptive statistics summarized the findings.

Results: All prescriptions included basic patient and prescriber information; however, only 87 (29%) stated a diagnosis, and 14 (4.7%) used generic drug names. The average number of drugs prescribed per prescription was 6.8 ± 1.7, with 213 (71%) of prescriptions involving polypharmacy (≥3 drugs). Antibiotics were prescribed in 137 (45.7%) of encounters, with complete adherence to the local antibiotic policy observed in 300 (100%) of the prescriptions. Injectable drugs were prescribed in 12 (4%) of the prescriptions. About 183 (61%) of prescribed drugs were from the National List of Essential Medicines, and 243 (81%) were available in the hospital pharmacy. Documentation of treatment duration and dosage schedules was present in 174 (58%) and 231 (77%) of prescriptions, respectively.

Conclusions: While basic prescription documentation meets standards, significant gaps remain in diagnosis recording, generic prescribing, and reduction of polypharmacy. Regular prescription audits and targeted educational interventions are recommended to enhance rational drug use and patient safety in the hospital.

## Introduction

A proficient approach to prescription writing is fundamental for healthcare professionals across diverse clinical settings. A clearly composed and comprehensive prescription not only effectively meets the therapeutic needs of patients but also plays a crucial role in minimizing medication errors during dispensing. Since a prescription serves as a legal document, it is essential for Registered Medical Practitioners to exercise diligence when issuing them. Medication errors may arise due to acts of commission or omission, potentially leading to significant consequences for prescribers, including loss of patient trust, legal challenges, and disciplinary actions by medical regulatory authorities. Globally, medication errors contribute to approximately 41% of hospital admissions, posing substantial social and economic burdens [1].

Several studies have shown that incorporating collaborative student-teacher sessions can help in enhancing the indispensable expertise of prescription writing at the student level. Incorporating diverse teaching methodologies such as P-drug exercises, problem-based learning activities, and prescription-writing tasks is an integral part of the medical curriculum. These educational strategies are instrumental in equipping learners with essential competencies to develop a rational medication inventory, select appropriate, case-specific drugs, and thereby enhance patient adherence. Thus, these approaches help reduce irrational and outdated prescribing practices in clinical settings [2].

The clarity and legibility of a prescription are equally crucial as are accurate diagnosis and selection of appropriate medications tailored to the patient's condition, all of which contribute to the overall quality and effectiveness of the prescription. Several studies have highlighted the grave economic and health impact of illegible handwriting of the doctors at the community level. Hence, it serves as an essential link between various factors that work together to deliver the optimal treatment to the patient [3].

A prescription analysis serves as an essential mechanism to enhance patient care and therapeutic outcomes by systematically reviewing clinical management. These audits systematically detect deficiencies and errors in prescribing practices. Comparing the prescriptions against the established standards helps the practitioner improve by identifying prescribing mistakes. This proactive approach encourages collective responsibility for patient care and quality. Therefore, it is imperative to perform regular audits separately for outpatient departments (OPD) and inpatient departments (IPD) to ensure adherence to the World Health Organization (WHO)’s core prescribing indicators [4]. Such audits will facilitate improvement in the quality and safety of medication use. Accordingly, this study was designed to assess the prescribing patterns, evaluate the completeness of prescriptions, identify deficient prescription components, and introduce the corrective measures to assist the prescribers in achieving higher standards of prescription quality.

## Materials and methods

This retrospective observational cross-sectional study was carried out under the Department of Pharmacology in association with the Department of Medicine, Hamdard Institute of Medical Sciences and Research (HIMSR), and associated Hakim Abdul Hamid Centenary Hospital (HAHCH) in South Delhi, Delhi, India. Before commencement of the study, we obtained approval from the institutional ethics committee, HIMSR, Jamia Hamdard, New Delhi (HIMSR/IEC/018/2021, dated March 19, 2021).

Sample size and sampling

Using the formula n = Z^2^ * P * (1 - P)/d^2^, the sample size was calculated to be a minimum of 145 at a 95% confidence level with a prevalence of 56.4%. Convenience sampling was used as a sampling technique. A total of 300 printed prescriptions were randomly sampled and analyzed using the hospital information system (HIS), irrespective of patient characteristics, diagnosis, and treatment [5]. The study data for prescription auditing were collected for a period of six months, i.e., August 2024-January 2025.

Data collection and analysis

The prescriptions from the Department of Medicine were accessed by logging in to the HIS, and relevant information like demographic data, diagnosis, drug information, and prescription completeness was collected on a preset prescription audit form manually. The collected data were screened, analyzed, and evaluated for appropriateness and rationality using the WHO core prescription indicators [6]. Manual entry of appropriate data into Microsoft Excel spreadsheets (Microsoft Corp., Redmond, WA, US) was performed on a day-to-day basis. Descriptive statistics were used to summarize the findings. The analysis of the OPD prescriptions encompassed three main categories: patient and prescriber information, drug-related information, and WHO core prescribing indicators.

## Results

A total of 300 printed prescriptions from the OPD of the Department of Medicine of a teaching tertiary care hospital in New Delhi, India, were systematically screened, collected, and evaluated. The standardized prescription format was a printed template that included the hospital name, patient’s full name, prescriber’s credentials, OPD registration number, and consultation date. Frequently observed omissions in the prescriptions involved precautionary instructions for patients regarding treatment, information about follow-up appointments, patient weight (in pediatric cases), and allergy history. The detailed results from both patient and prescriber data are presented in Table 1.

**Table 1:** Patient’s and prescriber’s information OPD: outpatient department

S. No.	Parameter	Number
1	OPD registration number mentioned	300 (100%)
2	Complete name of patient is written	300 (100%)
3	Age in years mentioned (months if <5 years)	267 (89%)
4	Weight in kg (only patients of pediatric age group)	48 (16%)
5	Gender of patient	300 (100%)
6	Date of consultation (day/month/year)	300 (100%)
7	Brief history written	243 (81%)
8	Salient features of clinical examination recorded	228 (76%)
9	Allergy status mentioned	5 (<2%)
10	Presumptive/definitive diagnosis written	87 (29%)
11	Investigations advised	141 (47%)
12	Follow-up advice and precautions are recorded	156 (52%)
13	In case of referral, the relevant clinical details and reason for referral given	165 (55%)
14	Date of next visit written	135 (45%)
15	Prescriber’s name	300 (100%)
16	Prescription duly signed	300 (100%)

Prescription analysis

The drug-related findings are presented according to the WHO core prescribing indicators. In this study, the mean number of drugs prescribed per prescription was 6.8 ± 1.7. Antibiotics were included in 69 (23.1%) of all prescriptions, while injectable medications accounted for 12 (4%) of the prescriptions. A total of 183 (61%) of drugs were prescribed from the National List of Essential Medicines (NLEM), and 14 (<5%) of drugs were prescribed by generic names (Table 2).

**Table 2:** WHO core prescribing indicators WHO: World Health Organization; NLEM: National List of Essential Medicines

S. No.	WHO indicators	Study value	WHO standards
1	Average number of drugs per prescription, mean (SD)	6.8 (±1.7)	1.6-1.8
2	Generic prescribing rate, %	14 (4.7%)	100%
3	Prescriptions with antibiotics, %	69 (23.1%)	20%-26.8%
4	Prescriptions with injections, %	12 (4%)	13.4%-24.1%
5	Use of essential medicines as per NLEM, %	183 (61%)	100%

**Figure 1:**
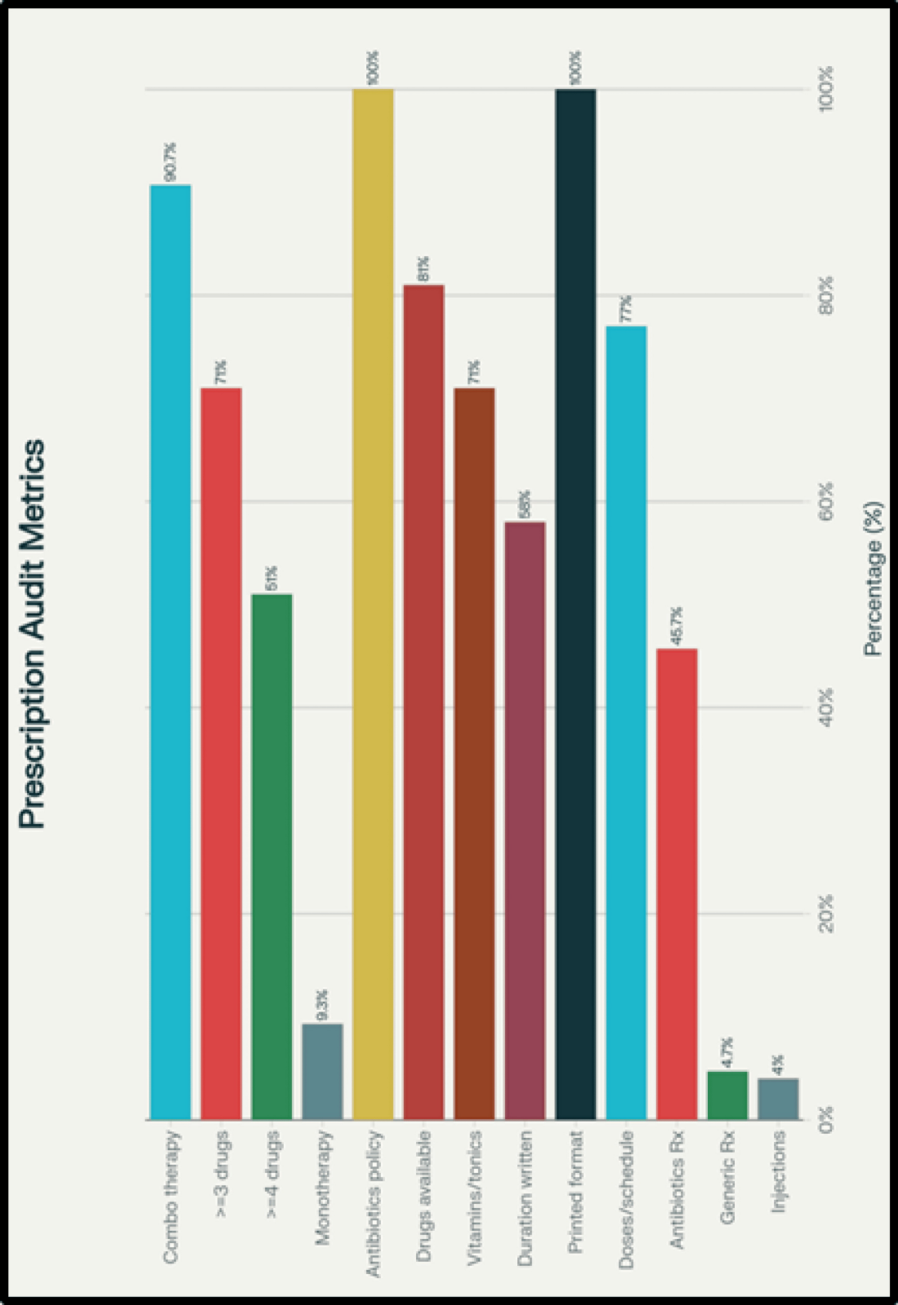
Drug-related information

All the prescriptions were in the printed format. Various multivitamin and multimineral formulations constituted more than 408 (20%) of all the prescribed medications. Over 1,428 (70%) of the prescribed medications were available at the hospital dispensary (Figure 1).

A comparison of the WHO core prescribing parameters between our study and similar studies done in the past is presented in Table 3.

**Table 3:** Comparison of the WHO core prescribing indicators with other studies WHO: World Health Organization; NLEM: National List of Essential Medicines

WHO core prescribing indicators	Our findings	Saha et al., 2018 [7]	Atal et al., 2021 [8]	Prasad et al., 2015 [9]
Mean no. of drugs	6.8	2.64	2.53	2.7
Prescription by generic name	14 (4.7%)	19.1%	16.0%	42.9%
Antibiotic use, %	69 (23.1%)	15.1%	19.8%	9.6%
Injection use, %	12 (4%)	1.2%	2.0%	1.6%
NLEM drugs prescribed, %	183 (61%)	52.9%	37.4%	95.6%

A total of 132 (44%) and 69 (23%) of the prescriptions lacked the total duration and the complete drug schedule, respectively. Findings are mentioned in Table 4.

**Table 4:** Comparison of indicators with other similar studies

Parameters	Our findings	Atal et al., 2021 [8]	Rai et al., 2018 [10]	Panayappan et al., 2017 [11]
Duration specified, %	168 (56%)	20.93%	36.1%	87%
Schedule documented, %	231 (77%)	11.63%	81.3%	85%
Diagnosis stated, %	210 (70%)	3.89%	56.6%	56%

Among the drugs prescribed, drugs inhibiting acid production constituted 288 (11.1%); antidiabetic medications constituted 388 (18.8%); antihypertensive medications constituted 307 (14.85%); non-steroidal anti-inflammatory drugs (NSAIDs) constituted 188 (9.1%), of which paracetamol (59 (31.2%)) was the most prescribed; antihyperlipidemic medications constituted 60 (2.9%); and various antimicrobials constituted 550 (26.6%) of the total drugs prescribed. Most common drugs prescribed from various classes have been summarized in Table 5.

**Table 5:** Commonly prescribed drugs by class NSAIDs: non-steroidal anti-inflammatory drugs

Drug class	Drug category	Number
Antacids/drugs inhibiting acid production	Pantoprazole	46 (2.2%)
	Pantoprazole + domperidone	57 (2.7%)
	Omeprazole	73 (3.5%)
	Ranitidine	56 (2.7%)
Vitamin and mineral preparations	B-complex vitamins	163 (7.9%)
	Vitamin D3	77 (3.7%)
	Calcium	63 (3.05%)
NSAIDs	Paracetamol	59 (2.8%)
	Aceclofenac	51 (2.4%)
	Naproxen	25 (1.2%)
	Ibuprofen	22 (1.06%)
	Etodolac	32 (1.5%)
Oral antidiabetic medications	Metformin	163 (7.9%)
	Glimepiride	84 (4.07%)
	Dapagliflozin	54 (2.6%)
	Vildagliptin	55 (2.6%)
Antihypertensive medications	Telmisartan	107 (5.1%)
	Amlodipine	83 (4.02%)
	Hydrochlorothiazide	57 (2.7%)
Antimicrobial medications	Cefixime	52 (2.5%)
	Amoxicillin-clavulanic acid	35 (1.6%)
	Azithromycin	27 (1.3%)
Antihyperlipidemic medications	Atorvastatin	33 (1.6%)
	Rosuvastatin	27 (1.3%)

Several fixed drug combinations (FDCs) were prescribed. The most prescribed FDCs in our study have been summarized in Table 6.

**Table 6:** Commonly prescribed combination therapy

Combination used	No. of prescriptions	NELM status
Pantoprazole + domperidone	57 (2.7%)	Not approved
Amoxicillin + clavulanate	35 (1.6%)	Approved
Etodolac + thiocolchicoside	31 (1.5%)	Not approved
Telmisartan + amlodipine	30 (1.45%)	Not approved
Telmisartan + cilnidipine	23 (1.1%)	Not approved
Glimepiride + metformin	63 (3.05%)	Not approved
Metformin + vildagliptin	52 (2.5%)	Not approved
Sitagliptin + metformin	28 (1.3%)	Not approved
Naproxen + domperidone	25 (1.2%)	Not approved
Pregabalin + methylcobalamin	35 (1.6%)	Not approved
Pregabalin + nortriptyline	32 (1.5%)	Not approved

As per the drug-utilization indicators, drug monotherapy was prescribed in only 28 (9.3%) prescriptions; the remaining 272 (90.7%) received combination therapy. Concomitant use of three or more drugs was prescribed in 213 (71%) of the patients, whereas four or more drugs were prescribed in 153 (51%) patients, suggesting a trend of polypharmacy (Figure 1).

The median duration of therapy was 8.2 days; a total of 168 (56%) patients received treatment for less than two weeks; 97 (32.3%) patients were prescribed pharmacotherapy for two to four weeks; and 35 (11.7%) patients were prescribed drugs for more than four weeks. Presumptive/definitive diagnosis was missing in 213 (71%) prescriptions. Rampant use of unapproved and obsolete short forms of drugs was widely prevalent, as was observed in several prescriptions. For example, the use of abbreviated terminologies like OD, BD, TDS, QID, HS, and BF was observed. The dose, frequency, duration of therapy, and route of drug administration were absent in many prescriptions.

## Discussion

Prescription audit is a fundamental tool in improving the quality of prescriptions of a healthcare facility. Prescription audits help in identifying important shortcomings and streamlining the specifics of standard care treatments that the patients receive from the hospital. The outcomes of this study provide important insights about the prescription patterns, common inclusions, and omissions in prescribing behavior.

All the prescriptions in our study were completely in printed format including the patient's and prescriber’s information, drug information, and instructions to the patient. Prescribing drugs in printed format helps in circumventing errors in prescribing practices due to sometimes cacographic handwriting of the prescribing physicians. As an important highlight in the existing literature, illegible handwriting of the prescribing physician irrefutably contributes to higher incidences of medication errors, which can have serious ramifications ranging from increased emergency visits and increased hospitalizations to deaths.

According to a survey, about 1.5 million people are harmed every year in the United States due to medication errors, with a substantial fraction of them resulting from misinterpretation of prescriptions. According to a US-based study conducted in 2006, about 7,000 mortalities annually are attributed as a direct linkage to medication errors [12]. Another similar study estimated illegible handwriting as a cause of up to 30,000 mortalities each year, a figure that highlights the criticality of this issue [12]. Although dispensing prescriptions electronically is more time-consuming, the advantages are unprecedented and unparalleled, and it is a giant leap in the domain of prescription writing [12].

The WHO core prescribing indicators are a set of vital benchmarks for assessing the suitability of drug usage in healthcare settings. The average number of medicines per prescription in our study was 6.8 ± 1.7, which was higher compared to other studies as well as the WHO recommendations, which suggests concomitantly prescribing more than two drugs per patient increases the likelihood of drug-drug and drug-food interactions, chances of non-adherence to treatment due to the increased pill burden on the patient, and increased cost of drug therapy. In a similar study done in the past by Prasad et al., the average number of drugs per prescription was 2.7, which is in contrast to our study [9].

Hence, prescribing multiple drugs and their combinations needs to be scrutinized to minimize adverse drug reactions (ADRs) and drug-drug interactions and enhance patient adherence. In our study, higher multivitamin, multimineral, and other drug formulations and enzyme prescriptions may have led to an overall increase in the pill count.

In our study, 183 (61%) of the drugs in all the prescriptions were prescribed from the NLEM. The use of drugs from the NLEM has been shown to improve healthcare and rational use of medicines when combined with proper procurement policies and good prescribing practices [13]. A similar study done in the past by Atal et al. reported 37% of all the drugs in the prescription from the NLEM [8].

The present study observed that 14 (<5%) medications were prescribed by their non-proprietary or generic name. A similar study done in the past by Atal et al. reported 16% drug prescription by generic name. Prescribing drugs by their generic name is of utmost importance as it promotes consistency and rational drug prescribing, avoids confusion caused by various brand names, limits the prescribers’ bias, and limits the cost of therapy. It also helps in limiting prescription errors by the identification of drug products by their universal scientific names and keeping a check on the prescription of look-alike sound-alike (LASA) drugs [8].

The present study observed a serious lapse in the documentation of the allergic history of the patients, with five (<2%) of the total prescriptions carrying the allergic history of the patients. A study from North India also reported similar limitations, with two (<1%) prescriptions carrying the allergic history of the patients [8].

A previous study reported >13,000 emergency visits due to drug allergy, of which 548 were anaphylactic events, and a total of 2,476 patients were hospitalized in the population group, thereby adding to the disease burden of the patient and society at large. Any given prescription should be standardized to record information about the drugs to reduce the chances of drug allergy. This practice also ensures that the patient and the family members are aware of the drugs or drug classes that they need to avoid and to check with the pharmacist before taking any over-the-counter medications [14].

Hence, taking proactive measures as simple as recording allergic history by virtue of active pharmacovigilance may help curtail the avoidable drug-induced allergic reactions. Although we are very short of our goal, we aim to achieve the WHO-recommended objective of 100% drug prescribing by their generic names as early as possible.

Various vitamins and minerals are one of the most commonly co-prescribed medications in the medicine clinics to cover up the unmet needs of nutritional deficiencies and various anti-inflammatory and wound healing properties [15]. In our study, B-complex vitamin was the most prescribed among the various multivitamins and minerals at 163 (7.9%), followed by vitamin D3 at 77 (3.7%). Calcium and vitamin D supplements were present in a significant number of prescriptions (3.05% and 3.7%), which is in concordance with a study done in the past [16]. Deficiency of calcium and vitamin D found extensively in the Indian population among almost all the age groups may have prompted the use of various calcium and vitamin D3 formulations [17]. However, easy over-the-counter availability and injudicious and rampant use of the health supplements without prior knowledge of the levels of these vitamins in the patients can lead to symptoms of hypervitaminosis, and hence, it remains a matter of grave concern.

Among the NSAIDs, paracetamol was observed to be the most prescribed, followed by aceclofenac and ibuprofen, which is in contrast with the study by Shankar et al., where diclofenac and meloxicam were the most prescribed NSAIDs [18]. Despite the extensive utilization of NSAIDs, their gastrointestinal side effect profile remains a major impediment to their clinical use. Hence, these drugs are mostly co-prescribed along with antacids/agents reducing acid production [19]. In the present study, we also observed the prescription of the gastroprotective agents along with NSAIDs. These findings are in communion with the study done by Nagla et al., where the proton pump inhibitors were the most common agents co-prescribed with analgesics [20].

The number of patients suffering from diabetes has been increasing in India, with a rise of almost 200% over the last two decades. In our study, 388 (18.8%) of the prescribed medications were oral antidiabetic medications. Similar studies have highlighted a similar distribution of disease in the community [21].

Similarly, there has been a rise in the number of hypertensive populations compared to the last two decades. In our study, 44 (14.8%) patients were on antihypertensive medications. These findings are similar to the study done in the past [22]. The prescribing physician should pay due cognizance while prescribing the two different classes of drugs concomitantly, as the combination may lead to the development of hypoglycemic episodes, for example, the use of combinations such as that of metformin with amlodipine, sulphonylureas with DPP-4 inhibitors, calcium channel blockers, etc.

In this study, we found that 55 (17.8%) drugs were prescribed in the form of FDCs. FDCs are a combination of one or more active ingredients to be used for a particular indication(s). The use of FDCs has been seen to improve patient adherence due to decreased pill burden, improvement of the treatment outcomes, and reduced cost [23]. However, the most neglected aspect associated with the use of FDCs is the occurrence of unanticipated side effects, extension of adverse effect profile, and emergence of drug resistance, if prescribed inadequately [23].

The trend of development and use is on the rise, especially over the last two decades, which has seen enormous production and consumption of a huge number of new and older drugs in the form of FDCs [24]. One of the major concerns associated with their use is the irrationality of the combinations.

The FDCs are rational only if they are pharmacokinetic-similar, the quality of the combination is non-inferior to the use of individual drugs, and there is augmentation of the efficacy as well as reduction in the incidence of adverse drug effects on combining the drugs [21]. One study highlighted that out of 264 FDCs available in the market, only 54 (20.5%) were deemed to be rational drug combinations. It was observed that while all the prescribing physicians were aware of the definition of FDCs, about 60% of them were unaware of the commonly prescribed FDCs being banned by the government of India (GOI) on account of being irrational/lacking scientific justification for FDCs [22]. ADRs were reported in about 14% of all patients, although a major fraction of the ADRs were of mild grade and did not necessitate any intervention.

In comparison to other similar studies, in our study, about 69 (23.1%) of the prescriptions contained antibiotics, which is similar to a previous study done by Saha et al., where 16% of the total prescriptions contained an antibiotic formulation. Judicious use of antibiotic formulations is imperative, as unnecessary use of antibiotic preparations can lead to the development of drug resistance [25]. Although within WHO guidelines, the proportion of injectable formulations among prescriptions in our sample was slightly higher than in comparable studies.

The study has certain limitations, including its single-center design, which may limit the generalizability of the findings to other settings. Additionally, the study relies on prescription data from a specific time frame, potentially overlooking seasonal variations in prescribing patterns. The absence of patient outcome measures and the lack of assessment of prescriber knowledge or behavior further constrain the comprehensive evaluation of prescription practices.

Implementing the audit recommendations, i.e., enhancing diagnosis documentation, reducing unnecessary polypharmacy, ensuring documentation of allergic history, and promoting generic prescribing, will reduce medication errors, prevent serious adverse events, improve treatment adherence, optimize therapeutic outcomes, improve healthcare accessibility, and reduce financial burden on patients. Future multicentric studies incorporating broader datasets and clinical outcomes are warranted to strengthen the evidence base.

## Conclusions

The prescription audit revealed critical gaps in rational prescribing practices within the tertiary care outpatient setting, particularly in diagnosis documentation, generic drug prescribing, and the prevalence of polypharmacy. While basic prescription elements such as patient and prescriber identification were adequately documented, omissions in allergy status, treatment duration, and medicine schedules were frequent. The high rate of antibiotic prescriptions underscores the urgent need for stringent antibiotic stewardship aligned with institutional policies. Implementing regular audit cycles coupled with focused educational interventions and feedback mechanisms is imperative to enhance prescription quality, ensure patient safety, and optimize resource utilization. Institutional efforts should aim at promoting generic prescribing, minimizing unnecessary polypharmacy, and standardizing prescription documentation to meet WHO core prescribing indicators.

Regular audit participation and professional development demonstrate commitment to quality improvement. Integrating these precautions into daily practice safeguards patient safety, maintains professional credibility, ensures medicolegal compliance, and fulfills the ethical obligation to deliver rational, evidence-based prescribing. This study supports the integration of prescription audits as a continuous quality improvement strategy in healthcare institutions.
